# Association between increased plasma levels of homocysteine and depression observed in individuals with primary lactose malabsorption

**DOI:** 10.1371/journal.pone.0202567

**Published:** 2018-08-23

**Authors:** Dietmar Enko, Andreas Meinitzer, Wolfgang Brandmayr, Gabriele Halwachs-Baumann, Wolfgang J. Schnedl, Gernot Kriegshäuser

**Affiliations:** 1 Institute of Clinical Chemistry and Laboratory Medicine, General Hospital Steyr, Steyr, Austria; 2 Clinical Institute of Medical and Chemical Laboratory Diagnostics, Medical University of Graz, Graz, Austria; 3 Department of Psychiatry and Psychotherapeutic Medicine, General Hospital Steyr, Steyr, Austria; 4 Practice for General Internal Medicine, Bruck/Mur, Austria; University of Illinois, UNITED STATES

## Abstract

**Background:**

Current literature proposes associations between homocysteine (HCY), folic acid (FA), vitamin B12 metabolism and depression. However, the exact underlying biological mechanisms remain unclear. This study aimed at evaluating a possible link between primary adult-type lactose malabsorption (PALM), HCY, FA and vitamin B12 metabolism and depressive disorder.

**Methods:**

Plasma levels of HCY, FA and vitamin B12 were determined in 78 patients with PALM and 160 individuals with lactase persistence sub-grouped by the presence or absence of major depression.

**Results:**

In 78 patients with PALM, the subgroup of 22 individuals with major depression showed significantly higher median (interquartile range) HCY (10.10 [8.46–12.03] vs. 8.9 [7.54–9.86] μmol/L, p = 0.029) and lower plasma FA levels (5.7 [4.68–9.14] vs. 6.95 [5.24–10.56] μmol/L, p = 0.272) compared to the subgroup of 56 individuals without depression, respectively. No such associations could be observed for those 160 individuals without PALM (i.e., lactase persistence)

Plasma HCY levels were positively correlated with depressive symptoms (p = 0.052), and showed negative correlations with FA (p = < 0.001) and vitamin B12 (p = 0.029), respectively.

**Conclusion:**

Depressed individuals with PALM were found with significantly higher HCY and lower FA levels compared to non-depressed individuals with PALM, however, this association was absent in the subgroup of lactase persistent individuals. These findings suggest an association between increased HCY levels, lactose malabsorption and depression.

## Introduction

Homocysteine (HCY) is a non-proteogenic thiol amino acid, which is generated from the methionine metabolism through demethylation [1]. The enzyme methionine-synthase catalyzes the reconstitution of HCY to methionine [1–3]. This biochemical reaction is mediated by the cofactors folic acid (FA) and vitamin B12, and reduced availability of these vitamins may result in increased HCY levels [4].

Recently published studies showed that elevated HCY concentrations are associated with depression [5, 6]. Moreover, elevated HCY levels have the potential to modulate neurotransmitters (e.g., dopamine, serotonin, melatonin) and dysregulate neurons, thereby increasing the risk of depression [7, 8]. Additionally, low FA plasma levels were found to be related with the diagnosis of major depression [9] and poorer clinical response to antidepressant treatment [7, 10]. Also, vitamin B12 was reported to have an inverse relationship with the risk of depressed symptoms [11, 12].

While current literature suggests these associations between vitamin B12, FA and HCY metabolism and depressive disorder, the nature of the relationship between elevated HCY blood levels and depression remains unclear [6]. There is some evidence, however, that increased HCY levels are the consequence rather than the cause of depression [13]. The question raises which underlying causative mechanisms influence the HCY metabolism in individuals with depression.

Currently malnutrition [14] and carbohydrate malabsorption [15] are proposed gastrointestinal conditions that lower the human FA status, thereby increasing the plasma HCY concentration. Moreover, Ledochowski et al. found lactose malabsorption linked to early signs of depression [16]. Primary adult-type lactose malabsorption (PALM) is an autosomal recessive gastrointestinal condition with declined lactase activity in the brush boarder of the small intestine [17–19]. Genotyping for the single-nucleotide polymorphism (C/T-13910) of the lactase gene (*LCT*) has been used to identify individuals with lactase non-persistence [18, 19]. The undigested non-absorbable lactose reaches the colon, where toxic bacterial degradation metabolites are formed. Interference of these microbial metabolic products with the neurotransmitter metabolism may be one explanation for the development of depression in individuals with PALM [16], however, the exact underlying biological mechanisms remain unclear.

Therefore, this study was conducted to evaluate possible associations between HCY, FA and vitamin B12 plasma levels in 78 patients with PALM and 160 individuals with lactase persistence sub-grouped by the presence or absence of major depression.

## Materials and methods

### Study design and patients

A total of 238 study participants were included in this prospective cohort study. The main demographic and clinical characteristics are summarized in Table 1. The median age was 41.5 (range: 16–70) years, and 66.0% of participants were female.

**Table 1:** Main demographic and clinical characteristics.

	Study population (n = 238)
**Demographic characteristics**:	
**Female (%)**	66.0 (n = 157)
**Male (%)**	34.0 (n = 81)
**Mean age (years)**	41.0 ± 14.4
**Anthropometric data**:	
**Height (m)**	1.7 (1.64–1.78)
**Weight (kg)**	73.0 (60.0–85.2)
**BMI (kg/m**^**2**^**)**	24.22 (21.63–28.09)
***LCT* C/T**_**-13910**_ **polymorphism**:	
**C/C**_**-13910**_ **homozygotes (%)**	32.8 (n = 78)
**C/T**_**-13910**_ **heterozygotes (%)**	42.8 (n = 102)
**T/T**_**-13910**_ **homozygotes (%)**	24.4 (n = 58)
**Psychiatric disorder**:	
**Major depression (%)**	33.6 (n = 80)
**Non depression (%)**	66.4 (n = 158)
**Depressive symptoms**:	
**BDI-II score**	9.0 (3.0–18.0)
**Laboratory parameters**:	
**Creatinine (mg/dL)**	0.72 ± 0.12
**eGFR (mL/min/1.73m**^**2**^**)**	108.7 ± 15.28
**Homocysteine (μmol/L)**	9.0 (7.9–10.3)
**Folic acid (ng/mL)**	6.7 (4.9–9.8)
**Vitamin B12 (pg/mL)**	398 (299.8–470.1)

BMI = body mass index; eGFR = estimated glomerular filtration rate; BDI-II = Beck Depression Inventory. Data are presented as percentage, means ± standard deviation, or medians (Q1 –Q3).

The study population consisted of 80 patients with major depression, diagnosed and treated at the Department of Psychiatry (General Hospital Steyr, Steyr, Austria), and 158 healthy individuals without depressive symptomatology or a former history of psychiatric disorder. All participants underwent genotyping for the lactase *LCT* C/T_-13910_ polymorphism and blood-sampling after an overnight fasting state in the morning evaluating plasma levels of HCY, FA and vitamin B12, and the kidney function (i.e., creatinine, estimated glomerular filtration rate [eGFR]). Additionally, the assessment of depressive symptoms was carried out with the self-rating Beck Depression Inventory (BDI-II) questionnaire [20]. Anthropometric data (weight, height and body mass index [BMI]) were determined using a calibrated personal scale and a wall-mounted metric tape.

None of the included patients had vitamin B12 or FA supplementation. Individuals with impaired renal function were excluded from the study.

All participants gave their written informed consent. This study was approved by the Ethical Committee of the Johannes Kepler University Linz (Linz, Austria) and carried out according to the latest version of the Declaration of Helsinki.

### Laboratory analyses

VACUETTE^®^ K3EDTA tubes (2 mL) (Greiner Bio-One International GmbH, Kremsmünster, Austria) were used for *LCT* C/T_-13910_ genotyping. Genomic DNA was extracted from 200 μL EDTA blood with the Nucleic Acid Isolation Kit I on the MagNA Pure Compact Instrument (Roche Diagnostics, Vienna, Austria) according to the manufacturer’s instructions. Genotyping for the *LCT* C/T_-13910_ polymorphism was performed by real-time PCR and melting curve analysis (*LCT* T-13910C ToolSet^™^ [Roche Diagnostics]) on a LightCycler 2.0 Instrument (Roche Diagnostics) [21].

Fasting blood samples were drawn into VACUETTE^®^ LH lithium tubes (4 mL) and centrifuged at 2000 x g for 10 minutes. HCY (normal range: 3.2–10.7 μmol/L), FA (normal range 3.1–17.5 ng/mL) and vitamin B12 (normal range: 182–625 pg/mL) measurements were performed by competitive chemiluminescent immunoassays on a Dimension Vista^®^ 1500 System (Siemens Healthcare GmbH; Vienna, Austria). Creatinine (male: 0.7–1.3 mg/dL; female: 0.55–1.02 mg/dL) was measured using an enzymatic method applied on a Roche Cobas Mira (Roche Diagnostics, Vienna, Austria). The eGFR (normal: >70 mL/min/1.73m2) was calculated applying the Chronic Kidney Disease Epidemiology Collaboration (CKD-EPI) equation [22].

### Statistical analysis

Descriptive statistics were used to present study variables. The Kolmogorov-Smirnov test was performed to calculate data distribution. Normally distributed continuous variables were given as means ± standard deviation (SD), and not normally distributed data as medians with interquartile ranges (Q1 –Q3). Categorical variables were expressed as percentages.

For subgroup comparisons of metric variables in case of normal distribution the independent two-sample Student’s *t*-test was used, in case of non-normal distributed variables, the exact Mann-Whitney *U*-test was calculated. Pearson’s correlation analyses were calculated to assess possible positive or negative correlations between HCY and other study parameters. A p-value of < 0.05 was considered statistically significant. For all statistical analyses, the Analyse-it^®^ software version 4.92 (Analyse-it Software, Ltd., Leeds, United Kingdom) was used.

## Results

### Study population

The observed frequencies for the *LCT* C/T_-13910_ polymorphism are given in Table 1. Seventy-eight out of 238 (32.8%) study participants could be identified as C/C_-13910_ homozygotes, the *LCT* genotype indicative of PALM. A total of 102/238 (42.8%) and 58/238 (24.4%) individuals were C/T_-13910_ hetero- and T/T_-13910_ homozygotes, respectively, with both genotypes indicating lactase persistence. All in all, 22/78 patients (28.2%) with PALM and 58/160 (36.3%) individuals with lactase persistence were diagnosed with major depression.

### HCY, FA and vitamin B12 measurements

No significant differences of median (interquartile ranges) HCY (9.15 [8.0–11.06] vs. 8.9 [7.79–10.20] μmol/L, p = 0.174), FA (6.75 [4.64–9.22] vs. 6.7 [5.09–10.32] ng/mL, p = 0.412), and vitamin B12 plasma levels (402.5 [301.4–467.2] vs. 396.5 [298.0–472.3] pg/mL, p = 0.848) were observed between 80 patients with major depression compared to 158 individuals without depression, respectively.

However, plasma HCY levels were positively correlated with the BDI-II score (p = 0.052, Pearson’s correlation coefficient [r] = 0.126) and creatinine (p < 0.001, r = 0.237) and showed a negative correlation with FA (p = < 0.001, r = - 0.313), with vitamin B12 (p = 0.029, r = -0.141), and with the eGFR (p = 0.001, r = - 0.205), respectively.

As shown in Table 2, 22 depressed patients with PALM had significantly higher median (interquartile range) HCY plasma levels (10.10 [8.46–12.03] vs. 8.9 [7.54–9.86] μmol/L, p = 0.029) compared to 56 non-depressed individuals with PALM. Interestingly, no significant differences of plasma HCY, FA and vitamin B12 concentrations were found for 58 depressed and 102 non-depressed individuals with lactase persistence (HCY: 8.9 [7.9–10.41] vs. 9.0 [7.8–10.51] μmol/L, p = 0.862; FA: 7.0 [4.6–9.33] vs. 6.5 [4.99–10.01] ng/mL, p = 0.798; vitamin B12: 377.0 [282.3–441.5] vs. 384.5 [297.9–470.8] pg/mL, p = 0.509), respectively.

**Table 2:** Comparison of study parameters for 22 depressed and 56 non-depressed individuals with primary-adult lactose malabsorption (PALM).

	Depression	Non depression	p-value
**Mean age (years)**	38.9 ± 12.2	39.3 ± 13.5	0.906
**Height (m)**	1.68 (1.65–1.78)	1.69 (1.62–1.74)	0.466
**Weight (kg)**	82.5 (64.6–92.1)	73.0 (61.0–79.0)	0.090
**BMI (kg/m**^**2**^**)**	26.49 (27.71–32.0)	24.19 (22.29–26.93)	0.168
**Creatinine (mg/dL)**	0.73 ± 0.13	0.72 ± 0.12	0.841
**eGFR (mL/min/1.73m**^**2**^**)**	108.42 ± 15.98	109.81 ± 14.04	0.705
**Homocysteine (μmol/L)**	10.10 (8.46–12.03)	8.9 (7.54–9.86)	0.029
**Folic acid (ng/mL)**	5.7 (4.68–9.14)	6.95 (5.24–10.56)	0.272
**Vitamin B12 (pg/mL)**	423.0 (386.8–470.4)	405.5 (308.3–474.3)	0.444
**BDI-II score**	28.0 (21.9–38.0)	5.0 (2.0–8.0)	< 0.001

BMI = body mass index; eGFR = estimated glomerular filtration rate; BDI-II = Beck Depression Inventory. Data are presented as means ± standard deviation or medians (Q1–Q3).

**Fig 1:**
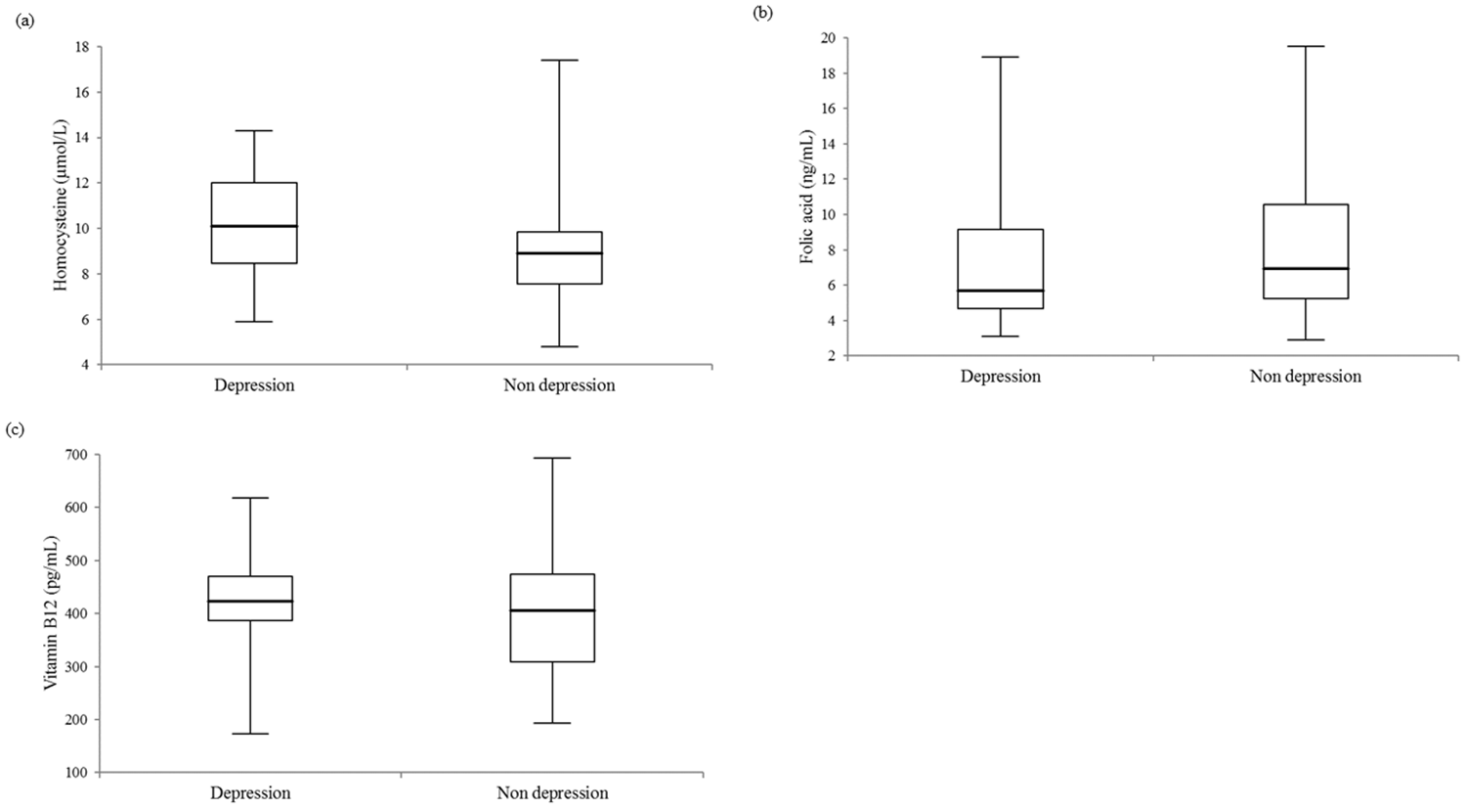
Box-and-whisker plots of (a) homocysteine, (b) folic acid, and (c) vitamin B12 plasma level comparisons between 22 depressed and 56 non-depressed individuals with primary-adult lactose malabsorption (PALM) (p-values were 0.029, 0.272, and 0.444). The central boxes represent the 25^th^ to 75^th^ percentile range. The lines inside the boxes show the median value for each group.

The box-and whisker plots of HCY, FA and vitamin B12 measurements for 78 individuals with PALM are illustrated in Fig 1(a)–1(c).

## Discussion

The current work investigated 78 subjects with PALM and 160 individuals with lactase persistence sub-grouped by the presence or absence of major depression for their HCY, FA and vitamin B12 plasma levels. To the best of our knowledge, this is the first study addressing a possible association between total plasma HCY and depression in individuals with PALM. We found patients with major depression and PALM to have significantly higher plasma HCY (10.10 [8.46–12.03] vs. 8.9 [7.54–9.86] μmol/L, p = 0.029) and lower FA plasma levels (5.7 [4.68–9.14] vs. 6.95 [5.24–10.56], p = 0.272) compared to non-depressed subjects with PALM. These associations, however, were not observed in individuals with lactase persistence. One possible explanation for this finding could be that carbohydrate malabsorption leads to changes in the bacterial colonization of the gastrointestinal tract [15]. Since the human intestinal flora is capable of synthesizing FA, which plays an important physiological role in the plasma FA concentrations and metabolism of healthy individuals [23], an altered bacterial composition in the gut could attribute to lower plasma FA levels in individuals with carbohydrate malabsorption [15]. In general, lower FA levels have been observed more frequently in patients with depression than in healthy controls [24, 25]. Changes in dietary habits may contribute to nutritional FA deficiency with decreased FA concentrations in these patients. [25, 26].

Elevated HCY plasma levels may occur as a result of decreased plasma FA concentrations because FA plays an important role as methyl-group donator for the conversion of HCY to methionine [27, 28]. Nevertheless, the diagnosis of functional FA deficiency may be uncertain by determining the plasma FA concentration alone because acute dietary high intake or restriction of this vitamin can influence its plasma levels independently of the intracellular stores [26]. Even the determination of red cell FA, which can be influenced by some analytical variables (e.g., hematocrit, oxygen saturation of hemoglobin), could not be confirmed as a better predictor of a patient’s FA status in clinical practice [29].

Current literature suggests total plasma HCY as a sensitive parameter to predict FA or vitamin B12 deficiency [26]. Regarding the present study population, plasma HCY levels showed a significant negative correlation with the FA (p = < 0.001, r = -0.313) and the vitamin B12 (p = 0.029, r = - 0.141) profile. This finding is in line with previous studies reporting an inverse relationship for these biomarkers [30, 31].

Here, plasma HCY levels correlated modestly with depressive symptoms (p = 0.052, r = 0.126). In comparison, other studies suggested a significant positive relationship for the severity of depressive symptoms and HCY concentrations [26, 32–34]. All these observations support the notion that there might be an association between HCY metabolism and depression. However, here we observed significant differences in median plasma HCY levels between subjects with and without major depression in the patient PALM subgroup, only. These findings indicate, that also other underlying pathophysiological factors, such as carbohydrate malabsorption, might be associated with elevated HCY levels and depression.

The major limitation of this cross-sectional study is the relatively low number of patients used to generate strong associations with biologically relevance. Furthermore, fructose malabsorption and dietary habits of study participants were not considered in the study design. Follow-up studies with large cohorts of patients comparing HCY, FA and vitamin B12 levels in a setting with and without different forms of carbohydrate malabsorption are warranted in order to fully elucidate possible associations between malabsorption syndromes, hyperhomocysteinemia and depression.

## Conclusions

Depressed individuals with PALM showed significantly higher HCY and lower FA levels compared to non-depressed individuals with PALM, however, this association was not observed in individuals with lactase persistence. These findings suggest a link between increased HCY levels, lactose malabsorption and depression.

## Supporting information

S1 Table The data set of the study is provided. (XLSX)

## References

[1] Obeid R, McCaddon A, Herrmann W. The role of hyperhomocysteinemia and B-vitamin deficiency in neurological and psychiatric diseases. Clin Chem Lab Med 2007; 45: 1590–1606. 10.1515/CCLM.2007.356 18067446

[2] Bottiglieri T. Homocysteine and folate metabolism in depression. Prog Neuropsychopharmacol Biol Psychiatry 2005; 29: 1103–1112. 10.1016/j.pnpbp.2005.06.021 16109454

[3] Reynolds EH. Folic acid, ageing, depression, and dementia. BMJ 2002; 324: 1512–1515. 12077044 10.1136/bmj.324.7352.1512 PMC1123448

[4] Shane B. Folate and vitamin B12 metabolism: overview and interaction with riboflavin, vitamin B6, and polymorphisms. Food Nutr Bull 2008; 29 (2 Suppl): S5–S16. 10.1177/15648265080292S103 18709878

[5] Li Y, Cao LL, Liu L, Qi QD. Serum levels of homocysteine at admission are associated with post-stroke depression in acute ischemic stroke. Neurol Sci 2017; 38: 811–817. 10.1007/s10072-017-2848-2 28215036

[6] Nabi H, Bochud M, Glaus J, Lasserre AM, Waeber G, Vollenweider P, et al Association of serum homocysteine with major depressive disorder: results from a large population-based study. Psychoneuroendocrinology 2013; 38: 2309–2318. 10.1016/j.psyneuen.2013.04.018 23707477

[7] Fava M, Borus JS, Alpert JE, Nierenberg AA, Rosenbaum JF, Bottiglieri T. Folate, vitamin B12, and homocysteine in major depressive disorder. Am J Psychiatry 1997; 154: 426–428. 10.1176/ajp.154.3.426 9054796

[8] Bhatia P, Singh N. Homocysteine excess: delineating the possible mechanism of neurotoxicity and depression. Fundam Clin Pharmacol 2015; 29: 522–528. 10.1111/fcp.12145 26376956

[9] Morris MS, Fava M, Jacques PF, Selhub J, Rosenberg IH. Depression and folate status in the US Population. Psychother Psychosom 2003; 72: 80–87. 10.1159/000068692 12601225

[10] Papakostas GI, Peterson T, Lebowitz BD, Mischoulon D, Ryan JL, Nierenberg AA, et al The relationship between serum folate, vitamin B12, and homocysteine levels in major depressive disorder and the timing of improvement with fluoxetine. Int J Neuropsychopharmacol 2005; 8: 523–526. 10.1017/S1461145705005195 15877935

[11] Seppälä J, Koponen H, Kautiainen H, Eriksson JG, Kampman O, Leiviskä J, et al Association between vitamin b12 levels and melancholic depressive symptoms: a Finnish population-based study. BMC Psychiatry 2013; 13: 145 10.1186/1471-244X-13-145 23705786 PMC3674945

[12] Ng TP, Feng L, Niti M, Kua EH, Yap KB. Folate, vitamin B12, homocysteine, and depressive symptoms in a population sample of older Chinese adults. J Am Geriatr Soc 2009; 57: 871–876. 19484842 10.1111/j.1532-5415.2009.02229.x

[13] Chengfeng S, Wei L, Xingxing W, Lei W, Rui Z, Lingjia Q. Hyperhomocysteinemia is a result, rather than a cause, of depression under chronic stress. PLoS One 2014; 9: e106625 10.1371/journal.pone.0106625 25286230 PMC4186820

[14] Frieling H, Römer KD, Beyer S, Hillemacher T, Wilhelm J, Jacoby GE, et al Depressive symptoms may explain elevated plasma levels of homocysteine in females with eating disorders. J Psychiatr Res 2008; 42: 83–86. 10.1016/j.jpsychires.2006.10.007 17182057

[15] Ledochowski M, Überall F, Propst T, Fuchs D. Fructose malabsorption is associated with lower plasma folic acid concentrations in middle-aged subjects. Clin Chem 1999; 45: 2013–2014. 10545075

[16] Ledochowski M, Sperner-Unterweger B, Fuchs D. Lactose malabsorption is associated with early signs of mental depression in females: a preliminary report. Dig Dis Sci; 43: 2513–2517. 9824144 10.1023/a:1026654820461

[17] Usai-Satta P, Scarpa M, Oppia F, Cabras F. Lactose malabsorption and intolerance: What should be the best clinical management? World J Gastrointest Pharmacol Ther 2012; 3: 29–33. 10.4292/wjgpt.v3.i3.29 22966480 PMC3437438

[18] Enattah NS, Sahi T, Savilahti E, Terwillinger JD, Peltonen L, Järvelä I. Identification of a variant associated with adult-type hypolactasia. Nat Genet 2002; 30: 233–237. 10.1038/ng826 11788828

[19] Enko D, Kriegshäuser G, Stolba R, Mangge H, Brandstetter D, Mayr N, et al Assessment of vitamin D status and serum CrossLaps levels in adults with primary lactose malabsorption. Eur J Clin Nutr 2016; 70: 1000–1003. 10.1038/ejcn.2016.66 27117931

[20] Steer RA, Ball R, Ranieri WF, Beck AT. Dimensions of the Beck Depression Inventory-II in clinically depressed outpatients. J Clin Psychol 1999; 55: 117–128. 10100838 10.1002/(sici)1097-4679(199901)55:1<117::aid-jclp12>3.0.co;2-a

[21] Enko D, Rezanka E, Stolba R, Halwachs-Baumann G. Lactose malabsorption testing in daily clinical practice: a critical retrospective analysis and comparison of the hydrogen/methane breath test and genetic test (c/t-13910 polymorphism) results. Gastroenterol Res Pract 2014; 2014: 464382 10.1155/2014/464382 24829570 PMC4009220

[22] Levey AS, Stevens LA, Schmid CH, Zhang YL, Castro AF 3rd, Feldman HI, et al A new equation to estimate glomerular filtration rate. Ann Intern Med 2009; 150: 604–612. 19414839 10.7326/0003-4819-150-9-200905050-00006 PMC2763564

[23] Camilo E, Zimmermann J, Mason JB, Golner B, Russell R, Selhub J, et al Folate synthesized by bacteria in the human upper small intestine is assimilated by the host. Gastroenterology 1996; 110: 991–998 8613033 10.1053/gast.1996.v110.pm8613033

[24] Morris DW, Trivedi MH, Rush AJ. Folate and unipolar depression. J Altern Complement Med 2008; 14: 277–285. 10.1089/acm.2007.0663 18370582

[25] Herrán A, García-Unzueta MT, Amado JA, López-Cordovilla JJ, Díez-Manrique JF, Vázquez-Barquero JL. Folate levels in psychiatric outpatients. Psychiatry Clin Neurosci 1999; 53: 531–533. 10.1046/j.1440-1819.1999.00572.x 10498238

[26] Bottiglieri T, Laundy M, Crellin R, Toone BK, Carney MW, Reynolds EH. Homocysteine, folate, methylation, and monoamine metabolism in depression. J Neurol Neurosurg Psychiatry 2000; 69: 228–232. 10.1136/jnnp.69.2.228 10896698 PMC1737050

[27] Paul RT, McDonnell AP, Kelly CB. Folic acid: neurochemistry, metabolism and relationship to depression. Hum Psychopharmacol 2004; 19: 477–488. 10.1002/hup.614 15378677

[28] Payne ME, Jamerson BD, Potocky CF, Ashley-Koch AE, Speer MC, Steffens DC. Natural food folate and late-life depression. J Nutr Elder 2009; 28: 348–358. 10.1080/01639360903417181 21184377 PMC3324853

[29] Farrell CJ, Kirsch SH, Herrmann M. Red cell or serum folate: what to do in clinical practice? Clin Chem Lab Med 2013; 51: 555–569. 10.1515/cclm-2012-0639 23449524

[30] Bjelland I, Tell GS, Vollset SE, Refsum H, Ueland PM. Folate, vitamin B12, homocysteine, and the MTHFR 677C->T polymorphism in anxiety and depression: the Hordaland Homocysteine Study. Arch Gen Psychiatry 2003; 60: 618–626. 10.1001/archpsyc.60.6.618 12796225

[31] Kim JM, Stewart R, Kim SW, Yang SJ, Shin IS, Yoon JS. Predictive value of folate, vitamin B12 and homocysteine levels in late-life depression. Br J Psychiatry 2008; 192: 268–274. 10.1192/bjp.bp.107.039511 18378986

[32] Tolmunen T, Hintikka J, Voutilainen S, Ruusunen A, Alfthan G, Nyyssönen K, et al Association between depressive symptoms and serum concentrations of homocysteine in men: a population based study. Am J Clin Nutr 2004; 80: 1574–1578. 10.1093/ajcn/80.6.1574 15585771

[33] Gu P, DeFina LF, Leonhard D, John S, Weiner MF, Brown ES. Relationship between serum homocysteine levels and depressive symptoms: the Cooper Center Longitudinal Study. J Clin Psychiatry 2012; 73: 691–695. 10.4088/JCP.11m07223 22480447

[34] Bender A, Hagan KE, Kingston N. The association of folate and depression: A meta-analysis. J Psychiatr Res 2017; 95: 9–18. 10.1016/j.jpsychires.2017.07.019 28759846

